# Abnormal myocardial perfusion correlates with impaired systolic strain and diastolic strain rate in systemic lupus erythematosus: a cardiovascular magnetic resonance study

**DOI:** 10.1186/1532-429X-17-S1-O81

**Published:** 2015-02-03

**Authors:** Ntobeko A Ntusi, Emily Sever, Joseph Lockey, Jane M Francis, Stefan K Piechnik, Vanessa M Ferreira, Paul M Matthews, Paul B Wordsworth, Stefan Neubauer, Theodoros D Karamitsos

**Affiliations:** 1 OCMR, Division of Cardiovascular Medicine, University of Oxford, Oxford, UK; 2 Division of Cardiology, Department of Medicine, University of Cape Town, Cape Town, South Africa; 3 Division of Brain Sciences, Imperial College London, London, UK; 4 Bortnar Institute, Nuffield Department of Orthopaedics, Rheumatology and Musculoskeletal Sciences, University of Oxford, Oxford, UK

## Background

Systemic lupus erythematosus (SLE) is a systemic autoimmune disorder that commonly affects the heart, resulting in a 7 to 9 times greater incidence of cardiovascular disease (CVD) in SLE patients compared to healthy controls. Female patients with SLE between 35 and 44 years old have an incidence of myocardial infarction over 50 times greater than that observed in the Framingham cohort. The clinical utility of cardiovascular magnetic resonance (CMR) first-pass perfusion for assessment of myocardial ischaemia is well-established. We hypothesised that CMR including stress first-pass perfusion would be able to detect coronary microvascular disease and subtle functional abnormalities in SLE and aimed to detect myocardial ischaemia in SLE using adenosine stress perfusion CMR.

## Methods

29 SLE patients (28 female, mean age 42 ± 9 years) and 29 matched controls (28 female, mean age 42 ± 9 years) without previously known cardiovascular disease underwent CMR at 1.5T including cine, tagging, T1 mapping, T2-weighted, perfusion imaging, late gadolinium enhancement (0.15mmol/kg gadoteric acid - Dotarem®) and ECV quantification. Comorbid status, disease activity index and duration of disease were recorded for each subject.

## Results

SLE patients were matched with controls for age, sex and comorbidity (Table 1). Myocardial perfusion reserve index (MPRI) was lower in SLE compared to controls (1.4 ± 0.2 vs. 1.9 ± 0.4, p<0.001), shown in Table 2. A third of lupus patients had visual evidence of non-segmental subendocardial perfusion defects, in keeping with microvascular dysfunction. No segmental perfusion defects were observed to suggest presence of epicardial coronary artery disease. There was no significant difference in LV size, mass and ejection fraction between SLE patients and controls. Peak systolic circumferential strain (-17.2 ± 1.7 vs. -19.4 ± 1.2, p<0.001) and peak diastolic strain rate (78 ± 24 vs. 118 ± 15 s^-1^, p<0.001) were impaired in SLE patients. In SLE, MPRI showed a significant correlation with peak systolic strain (R= -0.76, p<0.001) and peak diastolic strain rate (R= 0.65, p<0.001), depicted in Figure 1.

**Table 1:** Baseline characteristics of SLE patients and controls

	Controls N=29	SLE N=29	P value
Female sex, n (%)	28 (97)	28 (97)	1.00

Age, years	42 ± 9	42 ± 9	0.69

Hypertension, n (%)	0	0	-

Diabetes, n (%)	0	0	-

Hyperlipidaemia, n (%)	1 (3)	0	-

BMI, kg/m2	23 ± 3	28 ± 6	<0.001

Chloroquine, n (%)	N/A	20 (69)	-

Prednisolone, n (%)	N/A	14 (48)	-

Azathioprine, n (%)	N/A	8 (29)	-

Mycophenolate mofetil, n (%)	N/A	5 (17)	-

Methotrexate, n (%)	N/A	1 (3)	-

Leflunomide, n (%)	N/A	1 (3)	-

Rituximab, n (%)	N/A	1 (3)	-

HRT/OCP	9 (31)	9 (31)	1.00

SLEDAI (median, IQR)	N/A	9 (1-16)	-

ESR, mm/hr (median, IQR)	N/A	6 (3-12)	-

CRP, mg/L (median, IQR)	1 (1-1)	4 (2-9)	<0.001

Duration of SLE, years (median, IQR)	N/A	9 (-12)	-

Duration of DMARDs, years (median, IQR)	N/A	5 (3-8)	-

Continuous data are mean ± SD unless otherwise indicated. Categorical data are frequency (percent) unless otherwise indicated. BMI, body mass index; CRP, C-reactive protein; DMARD, disease modifying anti-rheumatic drug(s); ESR, erythrocyte sedimentation rate; HRT/OCP, hormone replacement therapy or oral contraceptive pill; IQR, interquartile range; SLE, systemic lupus erythematosus; SLEDAI, systemic lupus erythematosus disease activity index

**Table 2:** Myocardial structure, function and perfusion in SLE patients and controls

	Controls N=29	SLE N=29	P value
LVEDV indexed to BSA, ml/m2	80 ± 15	78 ± 12	0.71

LVESV indexed to BSA, ml/m2	22 ± 6	20 ± 6	0.23

LVEF, %	74 ± 5	72 ± 6	0.54

LV Mass indexed to BSA, g/m2	51 ± 10	48 ± 10	0.18

LA size, mm	26 ± 4	32 ± 5	<0.001

Mid SA circumferential strain	-19.4 ± 1.2	-17.2 ± 1.7	<0.001

Peak diastolic circumferential strain rate (s-1)	118 ± 15	78 ± 24	<0.001

Presence of LGE (%)	0	10 (43)	-

Volume fraction of LGE>2SD (%)	0	2.7 ± 0.3	-

Global myocardial T2 SI Ratio	1.5 ± 0.1	1.7 ± 0.3	0.001

Volume fraction of oedema by T2 (%)	0	17 (7-23)	-

Average myocardial T1, ms	959 ± 28	983 ± 30	0.01

Volume fraction of T1>990ms (%)	3 (1-4)	38 (23-50)	<0.001

ECV (%)	28.1 ± 2.9	30.6 ± 4.0	0.04

Rest RPP	7, 741 ± 1, 308	9, 028 ± 1, 669	0.002

Rest RPP	11, 474 ± 2, 110	13, 140 ± 2, 537	0.009

MPRI	1.9 ± 0.4	1.4 ± 0.2	<0.001

Proportion of non-segmental perfusion defects (%)	0	9 (31)	-

Continuous data are mean ± SD unless otherwise indicated. ECV, extracellular volume; LA, left atrium; LGE, late gadolinium enhancement; LV, left ventricle/ventricular; LVEDV, left ventricular end-diastolic volume; LVEF, left ventricular ejection fraction; LVESV, left ventricular end-systolic volume; MPRI, myocardial perfusion reserve index; RPP, rate pressure product; SA, short axis; SI, signal intensity, SLE, systemic lupus erythematosus

## Conclusions

Myocardial perfusion is impaired in patients with SLE with no known heart disease. In these patients, impaired MPRI was associated with abnormal myocardial deformation characteristics. It is likely that chronic disease activity and myocardial inflammation results in abnormalities in microvascular function which predate the development of myocardial functional derangements. CMR is an important tool for assessment of subclinical myocardial disease in SLE.

## Funding

This study was funded by investigator-led grants from Guerbet and GlaxoSmithKline.

